# Medication prescribing errors and associated factors at the pediatric wards of Dessie Referral Hospital, Northeast Ethiopia

**DOI:** 10.1186/1755-7682-7-18

**Published:** 2014-05-03

**Authors:** Abebe Zeleke, Tesfahun Chanie, Mirkuzie Woldie

**Affiliations:** 1 Department of Pharmacy, Wollo University, P.O. Box: 1145, Dessie, Ethiopia; 2 Department of Pharmacy, Jimma University, Jimma, Ethiopia; 3 Department of Health Services Management, Jimma University, Jimma, Ethiopia

**Keywords:** Medication errors, Prescribing errors, Dessie, Pediatrics

## Abstract

**Background:**

Medication error is common and preventable cause of medical errors and occurs as a result of either human error or a system flaw. The consequences of such errors are more harmful and frequent among pediatric patients.

**Objective:**

To assess medication prescribing errors and associated factors in the pediatric wards of Dessie Referral Hospital, Northeast Ethiopia.

**Methods:**

A cross-sectional study was carried out in the pediatric wards of Dessie Referral Hospital from February 17 to March 17, 2012. Data on the prescribed drugs were collected from patient charts and prescription papers among all patients who were admitted during the study period. Descriptive statistics was used to determine frequency, prevalence, means, and standard deviations. The relationship between dependent and independent variables were computed using logistic regression (with significance declared at p-value of 0.05 and 95% confidence interval).

**Results:**

Out of the 384 Medication order s identified during the study, a total of 223 prescribing errors were identified. This corresponds to an overall medication prescribing error rate of 58.07%. Incomplete prescriptions and dosing errors were the two most common types of prescribing errors. Antibiotics (54.26%) were the most common classes of drugs subjected to prescribing error. Day of the week and route of administration were factors significantly associated with increased prescribing error.

**Conclusions:**

Medication prescribing errors are common in the pediatric wards of Dessie Referral Hospital. Improving quick access to up to date reference materials, providing regular refresher trainings and possibly including a clinical pharmacist in the healthcare team are recommended.

## Introduction

Optimal pharmacotherapy is achieved when the right drug with the correct dosage and quality reaches the right patient at the right time [1]. However, medication error is common and preventable cause of iatrogenic injuries and may result in hospitalization, unnecessary diagnostic evaluations, unnecessary treatments, and death [2-5]. In 1999, an expert panel of the Institute of Medicine estimated that 44,000 to 98,000 people in the United States die each year as a result of medical errors, making medical error the sixth to ninth leading cause of death [6].

Every stage of the medication use process (storage, prescription, transcription, preparation, dispensation, and administration of a drug) is vulnerable to errors [7,8], but errors are most frequent and common during prescribing and administration [9].

Medication error is particularly common in hospitalized patients specially among those who require multiple forms of pharmacological therapies, elderly, critically ill and pediatric patients [10]. Incidence and consequences of medication errors are higher and potentially more harmful in the pediatric population than in th1e adult population [11]. For adults, the reported incidence of errors ranges from 1% to 30% of all hospital admissions, or 5% of orders written while in pediatrics the number reported was as high as 15.6% of orders [12].

Pediatric inpatients are groups of patients more prone to medication errors because of different factors; weight-based dosing, the need for stock medicine dilution, decreased communication abilities of children and the high vulnerability of young and critically ill children to injury from medication (have a minor physiological reserve; immature renal and hepatic systems to compensate medication errors) [11,13-15].

To ensure safety and quality of patient care preventing medication errors is important, and it can be easily done in the early stages of medication processing (prescribing and preparing the medication) but they are difficult in later stages [16]. American Society of Health System Pharmacists (ASHP) recognizes that medication errors can be minimized by assessing the medication use process, identifying inadequacies within systems, and developing interventions to correct the recognized deficiencies [17].

Up to the investigator’s knowledge studies in this area are scanty in Ethiopia and no previous research was done in Dessie referral hospital. Therefore, this study was designed to determine prevalence of medication prescribing errors and underlying causes at the pediatric wards of Dessie Referral Hospital, Northeast Ethiopia.

## Methods and participants

### Study area and design

A cross-sectional study was conducted from February 17 to March 17, 2012 at Dessie referral hospital. The hospital is located in Dessie Town, Northeast Ethiopia, 401 km northeast of the capital city, Addis Ababa. Dessie referral hospital is the only referral hospital in this part of the country, with catchment population of seven million. The Hospital has 200 beds and 165 health professionals. The pediatric wards have 52 beds and 12 health professionals (2 general practitioners, 1 pediatrician and 9 nurses).

### Study participants

All medication prescribing interventions to all pediatric patients who were admitted to the pediatric inpatient wards during the study period were included. Data on prescribing interventions were collected from patient Charts and prescription papers.

### Data collection procedures

Data on prescribed drugs were collected using a structured format from patient’s charts and prescription papers by two clinical pharmacists and one hospital pharmacist, who were oriented on how to extract the required data from patient charts. The contents of data collection format included patient demographics, diagnosis, diagnostic laboratory results, date of prescription, name of medication, dosage regimen (dose, frequency, dosage form, route of administration and duration) and type of prescribing error.

### Data processing and analysis

Data was edited, coded, entered into SPSS for windows version 16.0. Descriptive statistics was used to determine frequency, prevalence, means, and standard deviations. Prevalence of error was calculated per 100 orders, per 100 administrations and 100 patient days. The relationship between dependent and independent variables was examined using logistic regression model. Level of statistical significance was set at p-value of 0.05 and confidence interval of 95%.

### Ethical considerations

Approval and permission was obtained from the Institutional Review Board of College of Public Health and Medical Sciences of Jimma University. An official letter of cooperation was written from the Department of Pharmacy of Jimma University to the clinical director of the Dessie referral hospital. Data extraction from patient charts was conducted after obtaining written consent from the caretakers of the patients. All the data collection accessed in due course were kept confidential. To ensure confidentiality the names of patients were replaced with codes.

### Definition of terms

● Prescribing error: Implies deviation of medication prescribing from standard practices and includes inappropriate (incorrect) drug selection, wrong dose, wrong frequency, wrong route and wrong dosage form. In this study, prescribing errors were identified by comparison of prescribed drugs with “national standard treatment guideline” [18] and “pocket book of Pediatric Hospital Care in Ethiopia” [19].

● Rate of prescribing errors: In this study it was calculated as the sum of each type of prescribing error to the total number of drugs orders.

● Patient days: The total sum of days each patient stayed in the hospital.

● Medication order: it is one prescribed item

● Incorrect drug selection: Prescribing potentially interacting drugs, contra indicated drugs and known allergies.

● Wrong frequency: Drug prescribed with a frequency that deviates from the recommended practices.

● Wrong dose (prescribed): Dose of the drug ordered is ±10% of the recommended dose.

● Wrong route: Drug prescribed other than the recommended route.

● Incomplete prescription: Missing essential information in the prescription, such as missing the route of administration, the dose intended by the prescriber, frequency or type of dosage form.

● Antibiotics: In this study referred to antibacterial drugs.

## Results

### Characteristics of participants

During the 4 weeks, there were 150 admissions at the pediatric wards in Dessie Referral Hospital. Of these 136 admissions were included in this study and a total of 384 Medication order s were given for these patients over a period of 642 patient days.

Among the admitted patients majority were males (61.8%) and within the age group of 29 days – 3 years. On average each patient stayed 4.72 (±3.08) days and 27.9% of the patients were unconscious (Table 1). Three physicians (1 specialist, 2 general practitioners) were involved in medication prescribing.

**Table 1:** Characteristics of patients admitted in pediatric ward of Dessie referral hospital, February 17 - March 17, 2012 (n = 136)

**Patient Characteristics**		**Frequency (%)**
Age	Neonate (birth - 28 day)	22 (16.2)
	Infant (29 day - 1 year)	32 (23.5)
	Toddler (1–3 year)	32 (23.5)
	Preschool (3–5 years)	9 (−)
	School age (6–10 years)	22 (16.2)
	Adolescent (11–14 years)	19 (14.0)
Sex	Male	84 (61.8)
	Female	52 (38.2)
Level of consciousness	Conscious	98 (72.1)
	Not conscious	38 (27.9)

### Prevalence and nature of prescribing errors

**Figure 1:**
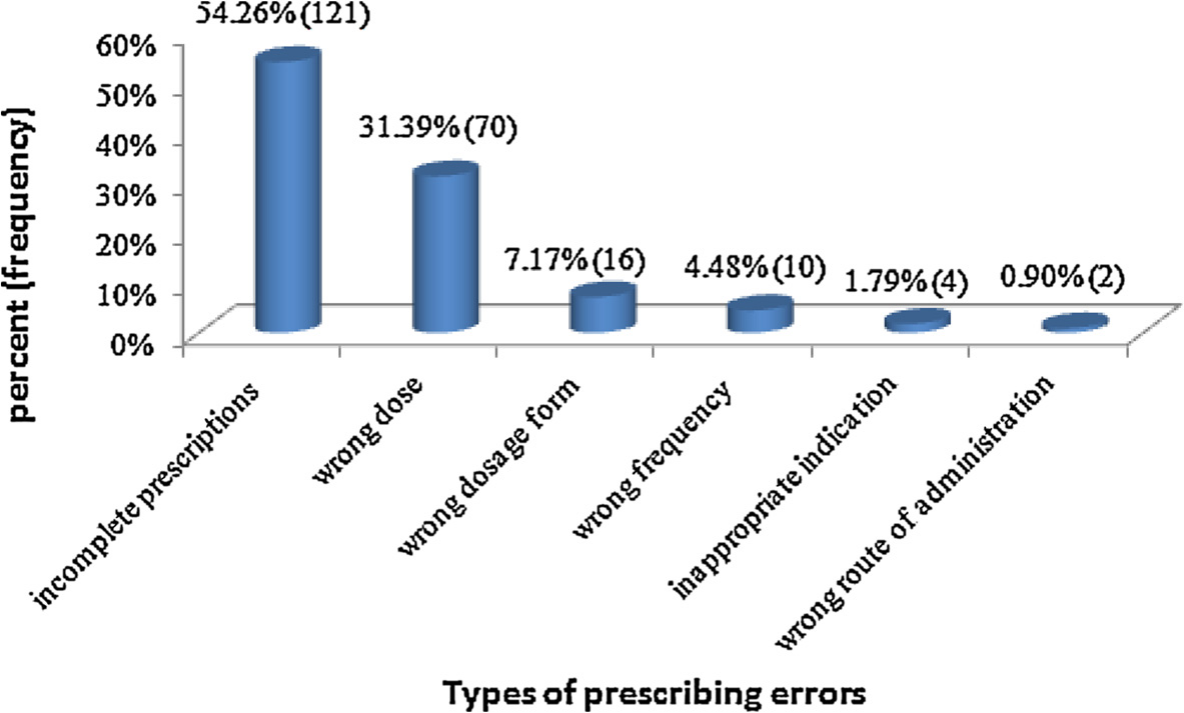
Types of medication prescribing errors at the pediatric ward of Dessie referral hospital, February 17 - March 17, 2012.

Among the 384 medication orders a total of 223 prescribing errors were identified. This corresponds to an overall medication prescribing error rate of 58.07% and 34.70 medication prescribing errors in 100 patient days. Incomplete prescriptions and dosing errors were the most prevalent error types which accounted for 54.26% and 31.39%, respectively. Figure 1 below provides an overall summary of the types of errors detected. Table 2 summarizes examples from each type of prescribing errors.

**Table 2:** Examples of medication prescribing errors in the pediatric ward of Dessie referral hospital, February 17 - March 17, 2012

**Type of prescribing error**	**Examples**
Wrong dose	A 3 year old patient ,weight 10 kg, was prescribed ceftriaxone 250 mg IV BID instead of the correct dose of 500 mg IV BID in the treatment of meningitis
Wrong route	Diclofenac sodium 75 mg was prescribed to be given through IV instead the correct route IM route
Incomplete prescriptions	Paracetamol suppositories: missing essential information about dose, frequency and route of administration
Inappropriate indication	Both Dexamethasone 8 mg IV stat and dexamethasone 8 mg IV QID for 3 days were prescribed for the treatment of severe croup, while dexamethasone 8 mg QID for 3 days was unnecessary
Wrong dosage form	Amoxicillin syrup 125 mg/5 ml was prescribed instead of the correct dosage form which is suspension
Wrong frequency	Ampicillin 30 mg intravenous was prescribed four times a day instead of the correct frequency of twice a day for a one day old baby

Drugs involved in prescribing errors were categorized into different therapeutic classes and antibiotics (54.26%) were the most prevalent categories. As individual drug paracetamol (13.00%) was most frequently associated with prescribing error. Tables 3 and 4 summarize different classes of drugs and top 10 most common drugs involved in prescribing error.

**Table 3:** Therapeutic category of medications associated with medication prescribing errors in the pediatric ward of Dessie referral hospital, February 17 - March 17, 2012

**Therapeutic class**	**Frequency (%)**
Antibiotics	121 (54.26)
Analgesics and antipyretics	41 (18.38)
Diuretics	10 (4.48)
Corticosteroids	3 (−)
GI drugs	2 (−)

**Table 4:** Top ten drugs associated with medication prescribing errors in the pediatric ward of Dessie referral hospital, February 17 - March 17, 2012

**Specific drug name**	**Wrong dose**	**Incomplete prescriptions**	**Wrong route**	**Wrong frequency**	**Wrong dosage form**	**Inappropriate indication**	**Total Frequency (%)**
Paracetamol	0	29	0	0	0	0	29 (13.00)
Amoxicillin	0	18	0	0	10	0	28 (12.55)
Ampicillin	16	2	0	6	0	0	24 (10.76)
Vitamin A	6	12	0	1	0	0	19 (8.52)
Gentamicin	15	1	0	2	0	0	18 (8.07)
Ceftriaxone	13	4	0	0	0	0	17 (7.62)
Crystalline penicillin	5	7	0	0	0	0	12 (5.38)
Diclofenac	1	9	1	0	0	0	11 (4.93)
Cotrimoxazole	0	4	0	0	3	0	7 (−)
Salbutamol	3	4	0	0	0	0	7 (−)

### Predictors of prescribing error

For exploring factors associated with increased risk of prescribing errors age of the patient, day of the week and route of administration were included in the bivariate analysis. The rate of medication error was twice in the age group 29 day – 1 year as compared to those patients who were 28 days old or younger (COR: 2.203; 95% [CI]: 1.116 – 4.347). Patients who were seen during weekends and holidays were about 50% less likely to encounter error as compared to those who were seen during weekdays (COR: 0.487); 95% [CI]: 0.259 – 0.913). Patients to whom the drug was ordered through oral route were about 8 times more likely to have error as compared to those who received intravenous route (COR: 7.696; 95% [CI]: 4.256 – 13.913). Moreover, patients who received Medication order s through other routes (rectal, intramuscular and topical ) were 5 times more likely to have error as compared to intravenous route (COR: 5.109; 95% [CI]: 2.672 – 9.767) (Table 5).

**Table 5:** Frequency and variables associated with prescribing errors at Dessie referral hospital, February 17 - March 17, 2012

**Characteristics**	**No. of PEs**	**No. without PEs**	**P-value**	**COR**	**95% CI**
**Age**			0.089		
Neonate (birth - 28 day)	22	30		1.000	(reference )
Infant (29 day - 1 year)	63	39	0.023	2.203	(1.116 – 4.347)
Toddler (1–3 year)	39	39	0.390	1.364	(0.673 – 2.765)
Preschool (3–5 years)	14	9	0.141	2.121	(0.779 – 5.777)
School age (6–10 years)	29	38	0.915	1.041	(0.500 – 2.164)
Adolescent (11–14 years)	28	34	0.760	1.123	(0.534 – 2.362)
**Route of administrations**			0.000		
Intravenous	84	157		1.000	(reference )
Oral	70	17	0.000	7.696	(4.256 – 13.913)
Others^a^	41	15	0.000	5.109	(2.672 – 9.767)
**Day of the week**					
Weekdays	178	158		1.000	(reference )
Weekends and Holidays	17	31	0.025	0.487	(0.259 – 0.913)

Note: ^a^Intramuscular, rectal, topical.

*Abbreviations:PEs* Prescribing Errors, *COR* Crude Odds Ratio, *CI* Confidence Interval.

All variables that were found to have a P-value <0.1 in the bivariate analysis were included in stepwise logistic regression. Age of the patient, day of the week and route of administration were included and days of the week and route of drug administration ordered remained to be predictors of medication prescribing errors. Goodness-of-fit vale of the model was 0.822. Hence, those patients who were seen on holidays had about 60% less chance of encountering medication prescribing errors as compared to those seen on weekdays (AOR: 0.418); 95% [CI]: 0.207 – 0.844). On the other hand, those patient who received orders with intravenous route had about 8 times more likelihood of encountering prescribing errors as compared to those who received orders with oral and other routes (AOR: 7.834; 95% [CI]: 4.305 – 14.257 and AOR: 5.467; 95% [CI]: 2.823 – 10.586, respectively) (Table 6).

**Table 6:** Variables associated with prescribing errors in pediatric inpatients (Multivariate Analysis)

**Variables**	**P-value**	**AOR**	**95% CI**
Day of the week			
Weekdays		1.000	(reference )
Weekends and holidays	0.015	0.418	(0.207 – 0.844)
Route of administration	0.000		
Intravenous		1.000	(reference)
Oral	0.000	7.834	(4.305 – 14.257)
Others^a^	0.000	5.467	(2.823 – 10.586)

Note: ^a^Intramuscular, rectal, topical.

*Abbreviations:AOR* Adjusted Odds Ratio, *CI* Confidence Interval.

## Discussion

This study was conducted with the intention of measuring the prevalence of medication prescribing errors and identifying predictors of errors in a resource limited setting. It was shown that 223 (58.07%) of the medication orders had some kind of medication prescribing error. Comparable proportion of medication prescribing errors were reported by an earlier study from Saudi Arabia (56%) [20], while the one from Egypt (78.1%) was much higher [21]. At this point it has to be noted that wrong time of administration of intravenous drugs was included in the definition of medication prescribing error in the study from Egypt while this was not the case in the current study. On the other hand the result in this study was higher than those reported by Folli et al. (4.9 and 4.5 errors per 1000 medication orders in two US hospitals) [22], Condren et al. (9.7% in Tulsa, U.S.A) [22] and Ghaleb et al. (13.2% in five hospitals, London) [23]. This difference may be ascribed to the differences in the hospital settings such as differences in training levels of prescribers, availability of support system and composition of health care team and difference in the definition of prescribing errors.

In this study, incomplete prescriptions, wrong dose, wrong dosage form and wrong frequency were the commonest types of medication prescribing errors. Similarly, incomplete prescriptions and wrong dosing were identified as the most common prescribing errors in other studies [20,23]. Errors due to incomplete prescriptions might not result in immediate danger but would be expected to delay receipt of medications [24]. On the other hand, dosing errors in pediatrics might result in toxicity due to over dose or sub therapeutic concentration and ineffective treatment.

It was revealed that the three most common drug classes involved in medication prescribing errors were antibiotics, analgesics and antipyretics and diuretics. Likewise, antibiotics were the most common classes of drugs involved in medication prescribing errors in previous studies from the UK [23] and USA [22,24]. It might be probably antibiotics were among the most common drugs prescribed. On the other hand, paracetamol, amoxicillin and Ampicillin were the three most common drugs involved in prescribing error. It was observed that almost all of medication orders of paracetamol and amoxicillin were incomplete prescriptions which explains the why these drugs are top on this list.

With regard to predictors of prescribing error, day of the week and type of route of administration were found to be independent predictors of medication prescribing error in pediatric wards of the Dessie Referral Hospital. Intravenous route was less likely to be associated with prescribing error. This may partly be explained by the fact that prescribers take extra care while determining dose, frequency and route of drugs give through intravenous route. Moreover, it was found that majority of prescriptions for oral and other routes were incomplete. Rates of medication errors in different age groups were not significant but the peak was in the age group 29 days – 1 year, comparable to reports from Spain [25] and U.S.A [22].

On the other hand, unlike the findings of de Muga et al. [25], medication error rate was high when patients were seen during weekdays as compared to weekends and holidays. This might be attributed to the difference in number of prescribers and admitted patients during weekdays, weekends and holidays. It was observed that few patients were admitted during weekends and holidays.

Interpretation of the findings reported here needs consideration of the limitations of the study. Since the study included patients admitted to a single hospital generalization of findings must be made cautiously. This study did not relate the clinical consequence of the medication prescribing errors observed.

## Conclusions

Medication prescribing errors were significantly high in the pediatric ward of Dessie referral hospital. Antibiotics were the most common class of drugs involved in medication errors. Day of the week and route of administration were independent predictors of medication prescribing error.

Physicians should take extra precautions when writing prescriptions (especially antibiotics) to pediatrics patients considering the consequences of errors in this group of patients. Development of system to report and prevent medication error in the pediatric wards is recommended. In the long term, development and implementation of a computerized physician order entry system, ward-based clinical pharmacists, and improving communication between health care workers in the hospital are recommended.

## Competing interests

The authors declare that they have no competing interests.

## Authors’ contributions

AZ was involved in the conception, design, analysis, interpretation, report writing and manuscript writing. TC and MW have been involved in the design, analysis, interpretation and the writing of the report. MW was also involved in the writing of the manuscript. All authors have read and approved the final manuscript.
